# A neural link between generosity and happiness

**DOI:** 10.1038/ncomms15964

**Published:** 2017-07-11

**Authors:** Soyoung Q. Park, Thorsten Kahnt, Azade Dogan, Sabrina Strang, Ernst Fehr, Philippe N. Tobler

**Affiliations:** 1 Department of Psychology I, University of Lübeck, Lübeck 23562, Germany; 2 Department of Neurology, Feinberg School of Medicine, Northwestern University, Chicago, Illinois 60611, USA; 3 Laboratory for Social and Neural Systems Research, Department of Economics, University of Zurich, Zurich 8006, Switzerland

## Abstract

Generous behaviour is known to increase happiness, which could thereby motivate generosity. In this study, we use functional magnetic resonance imaging and a public pledge for future generosity to investigate the brain mechanisms that link generous behaviour with increases in happiness. Participants promised to spend money over the next 4 weeks either on others (experimental group) or on themselves (control group). Here, we report that, compared to controls, participants in the experimental group make more generous choices in an independent decision-making task and show stronger increases in self-reported happiness. Generous decisions engage the temporo-parietal junction (TPJ) in the experimental more than in the control group and differentially modulate the connectivity between TPJ and ventral striatum. Importantly, striatal activity during generous decisions is directly related to changes in happiness. These results demonstrate that top–down control of striatal activity plays a fundamental role in linking commitment-induced generosity with happiness.

## Abstract (web summary)

Generous behaviour increases happiness, but the neural underpinnings of this link are unknown. Here, authors show that promising to be generous changes the neural response in the temporo-parietal junction, and that the connection between this region and the ventral striatum was related to happiness.

Human societies benefit from their members’ generous behaviour, such as donating to charity or volunteering one’s time. Generous behaviour is costly as it involves the investment of one’s own resources for the benefit of others. Nevertheless, generous behaviour is common and occurs even in situations in which reputation or the reinforcing experience of relieving a recipient’s distress are irrelevant. For these reasons, standard economic theory fails to explain generous behaviour. Research in the field of psychology suggests that a possible motive for generous behaviour is the increased happiness with which it is associated^1^,^2^,^3^,^4^. For example, Dunn *et al*.^4^ found that spending money on others predicted an increase in happiness. This finding was supported by experimental studies across cultures and ages showing that participants who spent money (or sweets) on others reported higher levels of happiness compared to those who spent money (or sweets) on themselves^1^,^2^,^3^,^4^. This is in line with the idea that generous behaviour is driven by the positive emotion (also termed warm glow) that it evokes^5^. Despite the obvious importance of this motivation for generous behaviour, we do not have a mechanistic understanding of the neural processes linking generosity and happiness.

In neuroimaging studies, generous behaviour and happiness have mostly been investigated separately. Other-regarding behaviour has been associated with activity in the temporo-parietal junction (TPJ). Specifically, altruistic choice, generous choice and overcoming egocentricity bias have been correlated with functional activity and structural properties of the TPJ^6^,^7^,^8^,^9^. Conversely, other studies have suggested that happiness, due to its connection to reward and pleasure, recruits reward-related brain areas such as the ventral striatum and the orbitofrontal cortex (OFC). For example, in one study in healthy participants, striatal activation correlated with self-reported happiness that had been induced by reward cues^10^. Recently, Rutledge *et al*.^11^,^12^ proposed a neural model according to which happiness is directly associated with striatal activation. However, the exact neural mechanisms through which generosity drives happiness remain unknown.

In the present study, we used functional magnetic resonance imaging (fMRI) to investigate how generosity is linked to happiness on the neural level. To induce generous behaviour, we used the powerful method of a public pledge^13^,^14^,^15^. First, we informed all participants that they would receive weekly monetary endowments. Participants in the experimental group were asked to commit to spending their endowment on others during the next 4 weeks, while participants in the control group were asked to commit to spending their endowment on themselves. Then, all participants performed an independent decision-making task, in which they could behave more or less generously while brain activity was measured using fMRI. At the beginning of the experiment and again after scanning, participants reported their subjective level of happiness^16^. We hypothesized that participants who had committed to spending their endowment on others would behave more generously in the decision-making task as well as self-report greater increases in happiness as compared to the control group. Importantly, we predicted that the neural link between generosity and happiness would involve functional interactions between brain regions engaged in generous behaviour (TPJ) and those mediating happiness (ventral striatum). The results confirmed our hypotheses. We found significantly higher levels of generous behaviour and happiness, as reflected by greater TPJ activity for generous choices and generosity-related connectivity of the TPJ with striatal happiness regions in the experimental group. We thus conclude that the interplay of these brain regions links commitment-induced generosity with happiness.

## Results

### Participants and experimental procedure

**Figure 1:**
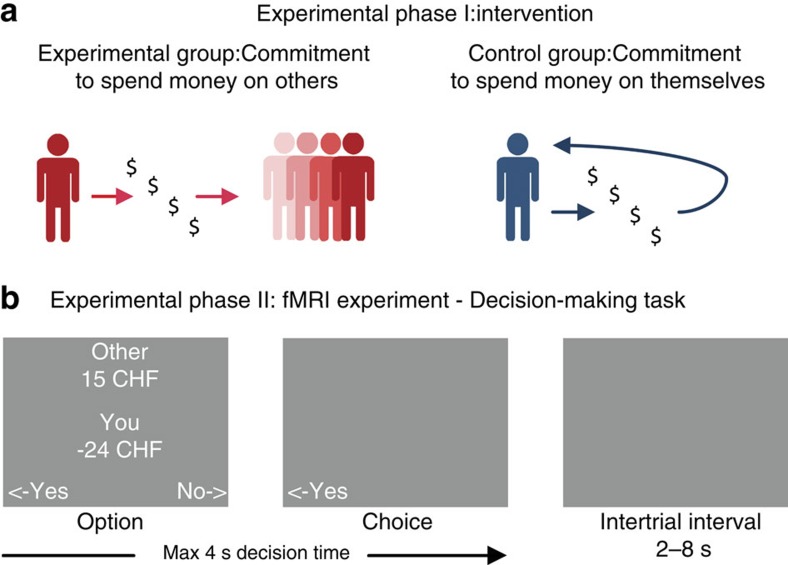
Experimental design. (**a**) First, we informed the participants that we would send them money for the next 4 weeks (25 Swiss francs per week). We asked half of the participants to commit to spending this money on other people (experimental group) and half of the participants to commit to spending the money on themselves (control group). In addition, we assessed the participants’ subjective happiness upon their arrival at the laboratory (T1), that is, before they had made the commitment, and after scanning, that is, at the end of the experiment (T2). (**b**) After the participants had made the commitment, they were asked to select one person to whom they wanted to give a present. Then they performed an independent decision-making task in the MRI scanner. In each trial, the participants were presented with an option that they could accept or reject. Each option was a combination of the benefits for the other person and the participants’ own costs. The magnitude of the benefits and costs varied independently and pseudorandomly from 3 to 25 Swiss francs. Each option was displayed for 4 s, which was the maximum response time. The selected response and the option were displayed together until the jittered ITI began. The participants responded by pressing the left or right arrow button, which corresponded to the ‘yes’ (accept) or ‘no’ (reject) displayed on the screen. The mapping between ‘yes’ or ‘no’ and left or right arrow buttons was randomized across trials.

The participants (*n*=50) were randomly assigned to either the experimental or the control group. In the first part of the experiment (Fig. 1a), we informed the participants that we would send each of them 25 Swiss francs in each of the following 4 weeks. We asked the participants in the experimental group to commit to spending the money on other people of their own choice. Examples included taking others out to dinner or buying gifts for others. We asked the participants in the control group to commit to spending the money on themselves. Examples included taking oneself out to dinner or buying oneself a gift.

After the participants had made a commitment, the second part of the experiment took place; the participants completed an independent decision-making task while we measured their blood-oxygen-level-dependent (BOLD) responses using fMRI (Fig. 1b). In the decision-making task, the participants first selected a person to whom they wanted to give a present (in the experimental group, the selected person was different from those the participant would spend money on in the following weeks). In each trial, the participants were presented with an option that they could accept or reject. Each option was a combination of monetary benefits for the other person and monetary costs to the participant. The magnitude of the benefits and the costs varied independently and pseudorandomly from 3 to 25 Swiss francs. All choice options included some costs, so that acceptance required at least some personal sacrifice on the part of the participant. Hence, we defined ‘generous behaviour’ in this task as the proportion of trials in which a participant accepted a personal cost to benefit someone else. In addition, we assessed individual happiness (using the subjective happiness scale, SHS^16^) as soon as the participant arrived at the laboratory (Time Point 1) and again after the participant had completed the decision-making task in the scanner (Time Point 2). This allowed us to determine the change in each participant’s self-reported level of happiness as a function of time (Time Point 2 versus Time Point 1) and group assignment (experimental versus control group). Furthermore, to rule out the possibility that familiarity with or affinity for the recipient named in the decision-making task had an impact on our measurement of generous behaviour, we asked the participants to rate how familiar they were with the recipient named in the decision-making task and how much they liked him or her. Two participants were excluded from further analyses because they had accepted all choice options (and rejected none) in the decision-making task, making a subsequent analysis of accept versus reject trials impossible. The remaining sample size was 48 (with 24 participants in each group).

### Behavioural effects of benefits and costs

**Figure 2:**
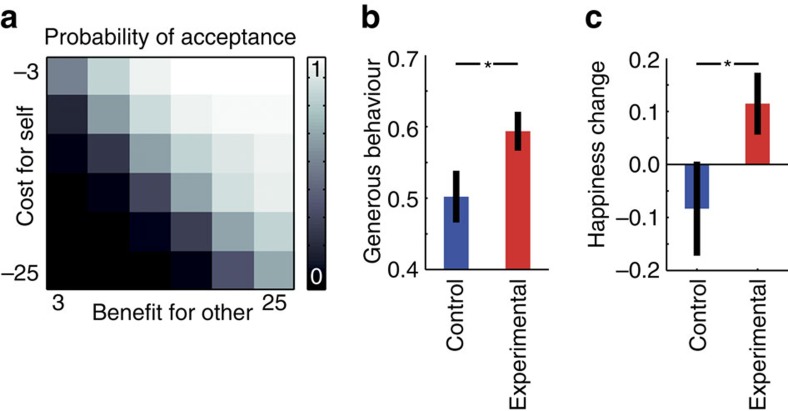
Behavioural data. (**a**) Mean probability of acceptance across all participants. The probability of acceptance increased as a function of the magnitude of benefits for the other and decreased as a function of the magnitude of costs to oneself. The benefits for the other and costs to oneself varied independently and pseudorandomly from 3 to 25 Swiss francs. (**b**) The participants in the experimental group, who had committed to spending the money on others, showed significantly more generous behaviour than the control group, who had committed to spending money on themselves. Generous behaviour was defined as the probability of accepting the option presented (*t*(46)=2.02; *P*<0.05). Error bars are s.e.m. (**c**) The participants in the experimental group showed a greater increase in happiness (Happiness(T2)-Happiness(T1)) than the control group did (*t*(46)=1.87; *P*<0.05). Error bars are s.e.m.

As a manipulation check, we first tested whether generosity increased as a function of the amount of monetary benefits for the other person and decreased as a function of the costs to the participant. As expected, across both groups, participants were more likely to behave generously as the amount of benefits for the other person increased and the costs to the participant decreased (Fig. 2a; average regression coefficient for other’s benefit; one-sample *t*-test, *n*=48, *b*=0.25, *t*(43)=17.2, *P*<0.001; own cost *b*=−0.22, *t*(43)=17.7, *P*<0.001). This effect was observable in each group separately and the coefficient for costs did not differ from the coefficient for benefits (experimental group; one-sample *t*-test, *n*=24, *t*(23)=1.72; *P*=0.1; control group; one-sample *t*-test, *n*=24, *t*(23)=0.42; *P*=0.68). The similarity of weight given to one’s own costs and to the other’s benefits could be due to the fact that the two were related and that the others were close to the participants, which may have reduced the amount of loss aversion experienced by the participants during the task.

A group comparison of the model parameters (weights) showed a significant main effect (ANOVA, *n*=48, *F*(3,138)=198.58, *P*<0.05) and a significant interaction (model parameters × group: *F*(3,138)=2.9, *P*<0.05). Post hoc *t*-tests revealed that neither the coefficient of cost (*t*-test, *n*=48, *t*(46)=0.72, *P*=0.477) nor of benefit (*t*(46)=−0.18, *P*=0.86) significantly differed, whereas the interaction coefficient (*t*-test, *n*=48, *t*(46)=−2.08, *P*<0.05) and the constant (*t*-test, *n*=48, *t*(46)=2.04, *P*<0.05) showed significant differences between the groups (Supplementary Table 1 for group mean±s.e.m.). The interaction coefficient was more negative in the experimental group than in the control group, indicating that experimental group participants were more likely than control group participants to accept offers in which costs and benefits were dissimilar, such as when recipients received a small benefit at a high cost.

### Control for familiarity and affinity

The two groups did not significantly differ with respect to their ratings of familiarity with and liking of the recipients (Kolmogorov–Smirnov test, *n*=48, familiarity: *P*=0.99; liking: *P*=0.65). Also, the familiarity and liking ratings did not significantly predict the individual differences in generosity (Kolmogorov–Smirnov test, *n*=24 for each group; experimental group: familiarity *r*=0.04, *P*=0.85; liking: *r*=0.16, *P*=0.46; control group: familiarity *r*=−0.12, *P*=0.58; liking: *r*=−0.02, *P*=0.93; all participants: familiarity *r*=−0.1, *P*=0.51; liking: *r*=−0.01, *P*=0.93). Therefore, we can rule out the possibility that the changes in generous behaviour described below were driven by group differences with respect to the familiarity with or liking of the recipient.

### Commitment to be generous increases generosity and happiness

Next, we assessed commitment-induced group differences in generosity and happiness. On average, the experimental group was more likely to make generous choices than the control group, as indicated by a significantly higher acceptance rate (that is, individual differences in generous behaviour; Fig. 2b; *t*-test, *n*=48, *t*(46)=2.02, *P*<0.05). Furthermore, participants in the experimental group reported a greater increase in happiness than did those in the control group (Fig. 2c; *t*-test, *n*=48, *t*(46)=1.87, *P*<0.05). However, generous behaviour and changes in happiness were not significantly correlated (Pearson correlation over all participants, *n*=48, *r*=0.2, *P*=0.16; experimental group: *n*=24, *r*=0.13, *P*=0.55; control group: *n*=24, *r*=0.16, *P*=0.46). Although it is difficult to compare results due to the differing study designs, this result is in line with that of a previous experimental study, namely, that participants reported being happier after behaving generously independent of the degree of generous behaviour displayed^4^. Thus, our behavioural results converge with those of the previous study in two aspects: (1) the increase in generous behaviour was concomitant to an increase in happiness and (2) the magnitude of the increase in happiness was independent of the increase in generosity^4^.

We performed numerous tests to rule out potential alternative explanations for the increases in generosity and happiness we observed. First, we considered the peak-end effect^17^, according to which outcomes towards the end of the experiment more strongly influence subjective experience than earlier outcomes. We therefore tested whether one of the groups received significantly better offers in the final trials and whether the final offers were related to increases in happiness. We did not find any significant group differences with respect to the options offered in the final trial (*t*-test, *n*=48, *t*=1.2, *P*=0.24), the mean over the final five trials (*t*-test, *n*=48, *t*=0.82, *P*=0.42), the mean over the final 10 trials (*t*-test, *n*=48, *t*=0.33, *P*=0.74), or the mean over the final 20 trials (*t*-test, *n*=48, *t*=0.59, *P*=0.56). Furthermore, none of these values predicted the increase in happiness (Pearson correlation; *n*=48, final trial: *r*=0.08, *P*=0.57; mean over the final five trials: Pearson correlation; *n*=48, *r*=0.11, *P*=0.46; mean over the final 10 trials: Pearson correlation; *n*=48, *r*=−0.01, *P*=0.96; mean over the final 20 trials: Pearson correlation; *n*=48, *r*=−0.03, *P*=0.82). Thus, it is unlikely that the peak-end effect played a major role in explaining the observed group differences in happiness.

We also assessed whether other variables could explain the behavioural effects and found that the two groups did not differ in trait empathy^18^ (*t*-test, *n*=48, all *P*’s>0.3, see Supplementary Table 2 for details) or in prosociality^19^ (*t*-test, *n*=48, *t*(46)=0.49, *P*=0.63). Moreover, we could not find a link between these variables and generous behaviour (Pearson correlation; *n*=48, all *P*’s>0.2; see Supplementary Table 3 for details). We also found no relationship between increase in happiness and inequality aversion as captured by Fehr-Schmidt model^20^ model (tested by two-sample *t*-test; *n*=48; no group differences in distaste for disadvantageous inequity (*t*=0.04, *P*=0.97) or in distaste for advantageous inequity (*t*=0.88, *P*=0.38)). Furthermore, we also found no link between inequality aversion and increase in happiness (tested by Pearson correlation; *n*=48, across groups: advantageous *r*=−0.06, *P*=0.67 disadvantageous *r*=−0.14, *P*=0.33; experimental group: advantageous *r*=−0.3, *P*=0.15 disadvantageous *r*=−0.09, *P*=0.67; control group: advantageous *r*=−0.16, *P*=0.47 disadvantageous *r*=−0.14, *P*=0.51). Thus, groups were well matched in terms of trait empathy, prosociality and inequality aversion, and these factors did not account for the effects of commitment on happiness and generosity.

### Commitment to be generous increases TPJ responses

**Figure 3:**
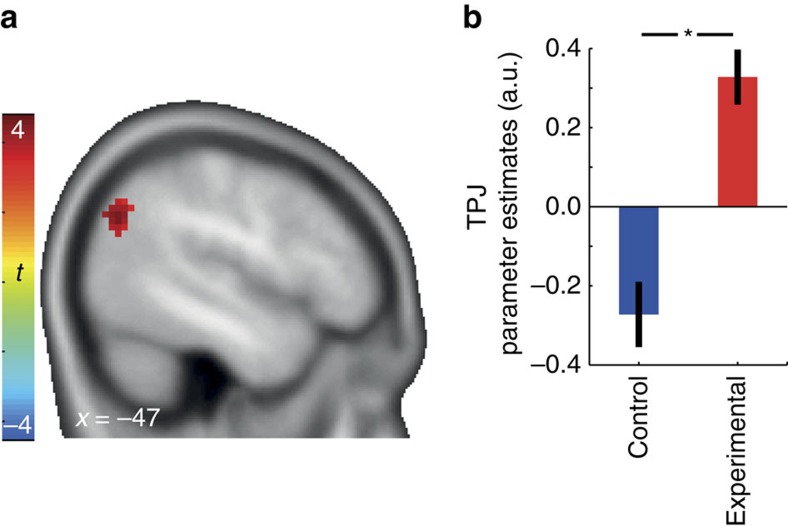
Commitment to be generous enhanced TPJ activity during decisions to be generous. (**a**) Compared to the control group participants, the experimental group participants showed significantly greater TPJ activation ((−51, −70, 34), *t*(46)=4.70) while accepting versus rejecting a personal cost to benefit another person. (**b**) Parameter estimates of the accept versus reject contrast, extracted from the TPJ region that showed significant group differences. Error bars are s.e.m.

First, we aimed to identify the general effects of the experimental manipulation (that is, commitment to generosity versus control commitment) on decision-related functional neural activity. We modelled BOLD activity at the time of decision separately for decisions to accept and to reject. We expected that a commitment to be generous would increase the recruitment of brain regions engaged in generous behaviour during such choices (that is, accept versus reject decisions). Indeed, a two-sample *t*-test revealed greater left TPJ activity in the experimental group than in the control group during generous choices (Fig. 3a,b, (−51, −70, 34), *n*=48, *t*(46)=4.70, *P*<0.05, whole brain family-wise error (FWE) corrected). The left TPJ was the only region that showed significantly greater activity in the experimental group than in the control group at this threshold (see Supplementary Fig. 1 for a bar graph depicting the left TPJ activation separately for accept and reject trials for each group; Supplementary Table 4 for whole-brain results; Supplementary Table 5 for correlation with behavioural generosity across groups), and there were no brain regions that showed greater activation in the control than in the experimental group.

Although only left TPJ activation survived the FWE-corrected threshold, our data do not support an argument for lateralization as a direct statistical comparison of left versus right hemisphere, with 3-mm or 5-mm spheres around the left peak coordinates, did not reveal a significant difference between left and right hemispheres (3-mm: *t*(47)=0.69, *P*=0.49; 5-mm: *t*(47)=0.98, *P*=0.33; paired *t*-tests; please note that testing at peak coordinates maximizes the chance for finding a difference; in line with this finding, an exploratory analysis at a more lenient threshold also revealed activity in right TPJ: Supplementary Fig. 2).

### TPJ connectivity reflects commitment-dependent generosity

**Figure 4:**
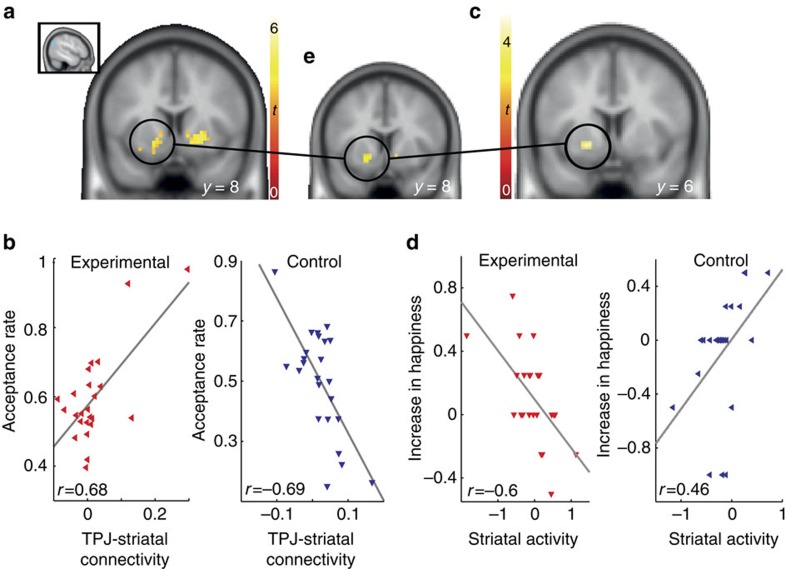
Striatum is modulated by TPJ as a function of generosity and tracks increase in happiness. (**a**) Striatal regions showing group differences in how generosity modulated connectivity with the TPJ. Inset illustrates TPJ seed region that was identified as the region showing significant group differences in accept versus reject trials (Fig. 3a). The psychological variable of the psychophysiological connectivity analysis was the contrast between accept versus reject trials. A group difference in TPJ-striatal connectivity was observable as a function of acceptance behaviour (right: (12, −1, −2), *t*(44)=5.81; left: (−15, 11, −5), *t*(44)=5.07; *P*<0.05, SV–FWE corrected). (**b**) We found a positive correlation between TPJ-striatal connectivity and generous behaviour in the experimental group; this correlation was negative in the control group. Thus, across participants, TPJ-striatum connectivity increased with generosity (defined as acceptance rate) in the experimental group, but decreased in the control group. Rank-based correlation analyses, which are robust against outliers, confirmed our results: Kendall’s tie-adjusted tau-b: 0.3, *P*=0.042, Spearman’s rho: 0.43; *P*=0.036. (**c**) Striatal region tracking group-dependent differences in coding increases in happiness during accept versus reject decisions ((−21, 2, −5) *t*(44)=4.34; *P*<0.05 FWE-corrected). (**d**) In the experimental group, we found a negative correlation between increase in happiness and striatal activity: The smaller the differences in striatal activity in accept as compared to reject trials, the greater the increase in happiness. In the control group, this correlation was positive. The ventral striatum activity in the two groups did not significantly differ (*t*(47)=1.68; *P*=0.1). Thus, the two groups differed with respect to how striatal activity predicted an increase in happiness, but not with respect to ventral striatum activity *per se*. (**e**) Conjunction analysis confirms that the same striatal region (1) tracks the increase in happiness and (2) is also modulated by TPJ connectivity as a function of generous behaviour.

Next, we examined the effects of commitment-induced increases in generosity-related TPJ activity on downstream brain regions. To this end, we performed a psychophysiological interaction (PPI) analysis (Methods section) using the TPJ as a seed region^21^,^22^. We hypothesized that activity in the ventral striatum and the OFC would be modulated by TPJ activity (accept versus reject) as a function of individual differences in generous behaviour and the type of commitment (self versus other). To test this hypothesis, we computed the group × acceptance rate interaction for TPJ connectivity during accept versus reject decisions. In line with our prediction, we found significant group differences in how connectivity between the TPJ and the ventral striatum was modulated by generous behaviour (Fig. 4a right: (12, −1, −2), *t*-test, *n*=48, *t*(44)=5.81; left: (−15, 11, −5), *t*(44)=5.07, *P*<0.05, small-volume family-wise error (SV—FWE) corrected; Supplementary Table 6 for whole-brain results; Supplementary Table 7 for analysis across groups). The TPJ-striatal connectivity was not modulated by the generosity commitment *per se* (*t*(47)=.311, *P*=0.76). Importantly however, the experimental and the control group showed different patterns of correlation between TPJ-striatal connectivity and generous behaviour (Fig. 4b). Specifically, greater TPJ-ventral striatum connectivity during accept versus reject trials was only associated with a greater acceptance rate in the experimental group, whereas this association was reversed in the control group. Thus, stronger TPJ-ventral striatum connectivity modulation facilitated generosity in the experimental group.

**Figure 5:**
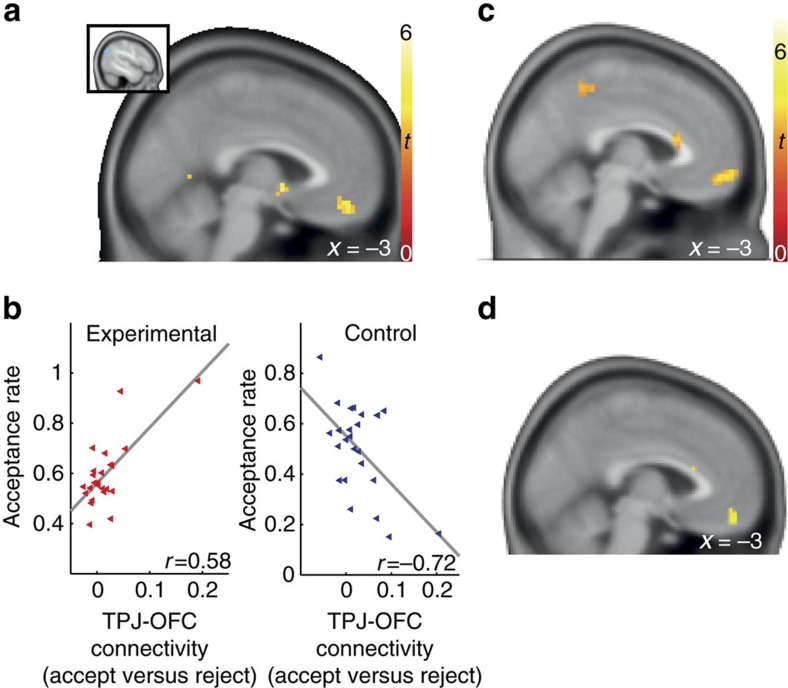
OFC codes subjective value of options and is modulated by TPJ. (**a**) TPJ-OFC connectivity reflecting group-dependent generosity (TPJ seed definition and PPI analysis as described in Fig. 4a). We observed a group difference in TPJ connectivity in a medial OFC region ((18, 38, −17), *t*(44)=5.60, *P*<0.05, SV–FWE corrected). Importantly, across participants, this connectivity was modulated by individual generosity (defined by acceptance rate) in a group-dependent manner. (**b**) In the experimental group, the participants who showed greater TPJ-OFC connectivity during the acceptance of an offer also showed more generous behaviour on average. In the control group, the opposite pattern was observed. Outliers-resistant rank-based correlation analyses confirmed the findings in the experimental group: Kendall’s tie-adjusted tau-b: 0.32, *P*=0.029, Spearman’s rho: 0.47; *P*=0.02. (**c**) We performed an additional analysis with trial-wise changes in subjective value as a parametric regressor. We identified an OFC cluster in which activity reflects the subjective value of the presented option in each trial ((0, 62, −11) *t*(45)=4.63; *P*<0.05, SV–FWE corrected). (**d**) Conjunction analysis confirms that the OFC region tracking subjective value is the very same region being modulated by the TPJ during acceptance decisions (conjuntion of **a**,**c**).

Given that the TPJ has been shown to modulate value signals in OFC during prosocial decisions^8^, we hypothesized that commitment-induced generosity would affect also interactions between the TPJ and value-coding OFC regions during generous decisions in our task. In line with this hypothesis, the PPI analysis showed that TPJ-OFC connectivity depended on both generosity and group (Fig. 5a,b; (18, 38, −17), *t*-test, *n*=48, *t*(44)=5.60, *P*<0.05, SV—FWE corrected; see Supplementary Table 6 for whole-brain results). Furthermore, we tested whether this OFC region also coded the subjective value of the decision option by integrating other’s benefit and own cost and found that it did (Fig. 5, and see section below entitled ‘TPJ modulates OFC region coding value of the choice option’).

### Ventral striatum activations predict changes in happiness

The above analyses show that the functional interaction between the TPJ and the ventral striatum is positively associated with generosity in the experimental group. We further predicted that the ventral striatum would play a key role in linking generous behaviour to happiness. We reasoned that activity in the striatum might link commitment-induced generosity and happiness by showing both (1) generosity-related and (2) happiness-related modulation, even though these two concepts have no significant shared variance. We therefore tested whether activity in the ventral striatum correlated with group-dependent changes in happiness. In other words, we tested the group × change-in-happiness interaction during accept versus reject decisions. We found that activity in the ventral striatum was significantly related to changes in happiness in a group-dependent manner (Fig. 4c, (−21, 2, −5) *t*-test, *n*=48, *t*(44)=4.34, *P*<0.05, SV—FWE corrected; Supplementary Table 8 for whole-brain results). Specifically, higher striatal activity during accept versus reject decisions was associated with greater increases in happiness in the control group (Fig. 4d). Conversely, in the experimental group, the participants with lower striatal activity reported greater increases in happiness. As a final step, we performed a conjunction analysis that confirmed the convergence of (1) group-specific generosity-predicting TPJ-ventral striatum connectivity and (2) group-specific coding of individual increases in happiness in the very same striatal region (Fig. 4e).

### TPJ modulates OFC region coding value of the choice option

The preceding analysis revealed no association between OFC activity and happiness (*t*-test, *n*=48, *t*(47)=−.825, *P*=0.414), suggesting that the OFC is not involved in linking generosity and happiness. However, it is conceivable that the OFC links generosity and subjective value. Therefore, we investigated the role of the OFC region that communicated with the TPJ in a group-specific manner. Since several studies have found that the OFC plays a role in coding the subjective value of choice options and social rewards, we conducted a parametric modulation analysis to test whether OFC activity codes the net subjective value of the presented choice option in each trial. We found that this was indeed the case: Over all participants, the OFC reflected the subjective value of the trial option (Fig. 5c; (0, 62, −11); *t*-test, *n*=48, *t*(45)=4.63, *P*<0.05, small-volume (SV)–family-wise error (FWE) corrected; Supplementary Table 9 for whole-brain results). Moreover, a conjunction analysis confirmed that the very same OFC region was also modulated by the TPJ in the connectivity analysis (Fig. 5d). In other words, the OFC region that coded the net subjective value of the option was functionally coupled with the TPJ in a manner that depended on commitment type and predicted generosity. Taken together, these results suggest that the generosity commitment modulated activity in the TPJ, which changed its connectivity with OFC coding the subjective value of the choices.

## Discussion

Economics, psychology, biology and philosophy have attempted to elucidate possible motives of generous behaviour. Proposed motives, such as helping kin, reciprocation or reputation, have limited explanatory power for the pervasive propensity of humans to be generous in different settings. One further explanation for generous behaviour is its link to happiness. The warm glow experienced when acting for the benefit of others has been proposed as a mechanism that reinforces generous behaviour in humans^5^. Our data suggest that a commitment to generous behaviour can increase happiness and thereby provide a neural mechanism that links commitment-induced generosity to happiness. Alternative factors, such as trait empathy, individual differences in prosociality and familiarity and liking of the recipient were not significantly correlated to generous behaviour. Furthermore, we were also able to rule out the peak-end effect as a potential explanation for our findings. Finally, we also tested for a link between inequality aversion and increase in happiness and found no relationship to inequality aversion as captured by the Fehr–Schmidt model^22^. However, our study was not specifically designed to identify such a link and a recent study by Rutledge *et al*.^23^ did reveal a link between inequality aversion and subjective happiness.

We find that a public pledge to be generous efficiently boosted generous behaviour and happiness in experimental relative to control participants, who had committed to spend money on themselves. The behavioural and neural changes induced by this method are striking, considering that participants had neither received nor spent any money at the time of the experiment. Public pledges are widely used to motivate future behaviour^14^,^15^, (Supplementary References 2 and 3). Also, in laboratory experimental settings, it has been shown that precommitment influences behavioural and neural self-control mechanisms^13^. On the psychological level, precommitment could induce a preference for avoiding inconsistency, leading people to act in line with their past or committed behaviour^24^,^25^. Yet, it is important to keep in mind that the opposite effect, the so-called moral licensing effect, has been observed as well. With moral licensing, past good behaviour increases subsequent immoral behaviour^26^,^27^. The critical factors leading to one or the other effect are still under debate^26^. One factor potentially enhancing the efficiency of commitment is framing: When initial goal pursuit is framed as commitment, people show commitment-consistent behaviour; framing it as progress towards the goal appears to facilitate licensing behaviour (ref. 28 Study 3). At the time of the experiment, our participants had not yet behaved according to their commitment (note that the beneficiary named in the scanning session and those the participant would spend money on in the subsequent behavioural implementation differed), which may have counteracted moral licensing. Our results demonstrate that a public pledge can be used as commitment strategy motivating commitment-consistent generous behaviour, which not only has an impact on generous decision making, but also on happiness.

Several studies have demonstrated changes in happiness induced by generous behaviour in both field and experimental settings^1^,^2^,^3^,^4^. Our study shows that even in a strictly controlled laboratory setting involving decision making in the MRI scanner, commitment induces generosity along with increases in happiness. The commitment changed not only TPJ responses, but also TPJ connectivity with striatum and OFC. Our results were specific to the experimental group, and collapsing across groups did not reveal any significant results. Interestingly, changes in happiness were driven by the commitment to be generous as such, independent of the absolute monetary amount spent on others. That is, the individual degree of generosity did not predict the individual changes in happiness in our study. This confirms previous findings, for example, from Dunn *et al*.^4^, who also reported that the amount of money spent on others was not directly related to changes in happiness.

The experimental group showed greater left TPJ activation than the control group when participants made generous decisions. Previous research has linked activity in the TPJ to social cognition^7^,^29^. For instance, theory-of-mind (ToM) and empathy paradigms activate the TPJ^30^,^31^. Another brain region that has often been found to be activated in this context is the dorsomedial prefrontal cortex (DMPFC^32^,^33^,^34^,^35^). Recent studies have suggested slightly different roles for the two regions. DMPFC may be preferentially involved in the integration of different emotions whereas the TPJ may be particularly involved in evaluating others’ emotions^36^, which could explain its role in prosocial behaviour. Indeed, across different paradigms, the TPJ showed increased activity especially when participants chose to forgo their own rewards in favour of rewards for others, which was not the case for DMPFC^6^,^8^,^37^. We found that commitment primarily affected generosity-related TPJ activity in the left hemisphere. The literature about TPJ effects in social tasks is mixed with regard to lateralization. For example, direct current stimulation during social tasks shows bilateral effects^38^. Whereas the functional neuroimaging literature often focuses on the right TPJ, meta-analyses of fMRI data in social tasks also report left-dominant effects^39^. The fact that, in our task, activity did not differ significantly between hemispheres is in line with the ensuing view that both left and right TPJ contribute to social functions.

Our findings suggest that TPJ activity as such is predominantly associated with generous behaviour^6^,^7^,^8^,^40^. Indeed, recent evidence suggests that the TPJ plays a crucial role in overcoming one’s own selfish motives when generosity is costly for oneself^41^. Our data are in line with this interpretation. Since generous behaviour in our paradigm included costs to the participant, generous behaviour always required overcoming selfish motives. Extending previous studies, our data show that TPJ activity can be enhanced by a public pledge to behave prosocially. Thus, compared to participants in the control group, participants in the experimental group were more effective in recruiting the TPJ and overcame their selfish response tendency to a larger extent, which resulted in more generous behaviour.

Previous studies have reported an association between generous behaviour and activity in reward-related regions^8^,^40^,^42^,^43^. For example, the OFC has been shown to be involved in computing the value of the option to donate to a charity^40^ and in coding the value of generosity decisions^8^. In both of these studies, the OFC changed its connectivity with the TPJ. Our results concur in that we find trial-by-trial decision values to be reflected in OFC activity and this activity to be modulated by TPJ connectivity.

In line with numerous reports across species^44^, our results show that the OFC represents the subjective value of choice options. Previous studies also found the OFC to be engaged during charitable donations^40^, social rewards^45^,^46^ and equitable interpersonal decisions^47^. Thus, the OFC does not exclusively represent one’s own reward values but incorporates other-regarding preferences in its value computation. Recent research suggested that this modulation takes place by a modulation from the TPJ^8^. Our data confirm this modulation of OFC activity by the TPJ. More specifically, we were able to show that greater TPJ-OFC connectivity predicts enhanced generous behaviour in the experimental group, suggesting that the TPJ regulates activity in the OFC and thereby increases the value of generous behaviour.

Our results demonstrate that the TPJ modulates activity in the ventral striatum and that this predicts generous behaviour in a group-dependent manner. As discussed above, the TPJ has previously been shown to modulate value-related activity in the OFC during generous choice^8^. Our findings suggest that the TPJ also functionally interacts with the ventral striatum and that this interaction is directly linked to commitment-induced generous behaviour. Importantly, our data further show that striatal activity during generous choices is predictive of increases in happiness. An interesting question arises whether the increase in happiness is driven by the generosity commitment *per se* or by generous behaviour during the experiment. Although, in line with previous studies^1^,^3^,^4^, we do not observe a behavioural link between the increase in happiness and generous behaviour, our neural data provide such a link in that the striatal region that codes the increase in happiness is also modulated by TPJ connectivity in a generous-behaviour-dependent manner.

In the control group, greater striatal activity during accept versus reject choices was associated with greater happiness. This is in line with previous evidence showing that momentary happiness is reflected in striatal activity^11^,^12^, which encouraged our a priori hypothesis that the ventral striatum would be the primary region for the link between generosity and happiness. In line with these observations, previous studies have shown that ventral striatum activity can reflect both rewards for the self and for others^42^,^43^,^48^.

In contrast, the opposite pattern was observed in the experimental group. Here, surprisingly, a lower level of striatal activity during accept versus reject choices was linked to greater happiness. One possible interpretation for this finding is that people who have committed to be generous at a later time are more willing to sacrifice short-term hedonic gain for themselves (as assessed trial-by-trial) for longer-term happiness (as assessed at the very end of experiment). This could explain why participants in the experimental group showed lower trial-by-trial ventral striatum activation during accept versus reject decisions, but reported greater happiness at the end of the experiment. Alternatively, it is possible that the discrepancy between our results and previous findings (for example, refs 42, 43) arises because each of the choices in the present study involved a balance of rewards for self versus others, which implicitly pits self-interest against other-regarding motives. In contrast, in the other studies, choices to give to oneself versus others were separated. Thus, it may be possible to harness ventral striatum activity for other-regarding ends, particularly when benefits for self and others are independent of each other and considered separately. Conversely, striatal lesions resulting in striatal hypoperfusion can result in so-called pathological generosity and personal ruin^49^, suggesting that the striatum may tip the balance towards self-interest in situations in which generosity comes at a personal cost.

Generosity and happiness improve individual well-being and can facilitate societal success. However, in everyday life, people underestimate the link between generosity and happiness and therefore overlook the benefits of prosocial spending. When asked, they respond that they assume there would be a greater increase in happiness after spending money on themselves and after spending greater amounts of money^4^. Our study provides behavioural and neural evidence that supports the link between generosity and happiness. Our results suggest that, for a person to achieve happiness from generous behaviour, the brain regions involved in empathy and social cognition need to overwrite selfish motives in reward-related brain regions. These findings have important implications not only for neuroscience but also for education, politics, economics and health.

## Methods

### Participants

Fifty healthy, right-handed participants (11 males; 25.6±0.7 years old, mean±s.e.m.) with normal or corrected-to-normal vision participated in the experiment. Two participants were excluded because they accepted all choice options in the decision-making task, making it impossible to compare brain measures for accept versus reject decisions. The remaining sample size was 48, 24 in each group. The study was approved by the Research Ethics Committee of the Canton of Zurich, and all participants provided informed consent.

### Experimental procedure

Upon arrival at the laboratory, the participants completed the subjective happiness scale questionnaire (SHS; ref. 16). Then the participants were randomly assigned to either the experimental or the control group. We informed the participants that we would send each of them 25 Swiss francs in each of the following 4 weeks (100 francs total). We asked the participants in the experimental group to commit to spending the money on other people of their own choice; examples included taking others out to dinner or buying presents for others. Participants were completely free to choose their recipients. We asked the participants in the control group to commit to spending the money on themselves; examples included taking oneself out to dinner or buying oneself a present. All participants made a commitment and none of them knew about the existence of the other group. After making a commitment, all of the participants performed an independent decision-making task in the MRI scanner (see section below entitled ‘Decision-making task’). Finally, subjective happiness was assessed a second time and the participants completed the social desirability scale (SDS; ref. 50).

### Decision-making task

All participants completed the decision-making task while we measured BOLD activity using fMRI. The participants first selected a person to whom they wanted to give a present. In the experimental group, this person was to differ from those the participants would spend money on in the subsequent weeks. Participants rated their familiarity with and liking of the recipient. In each trial, the participants were presented with an option that they could accept or reject. The option was a combination of monetary benefits for the other person and costs to the participant. For example, to be able to give a present worth 18 Swiss francs, the participant would have to pay the price of 25 Swiss francs. The benefits and costs varied independently and pseudorandomly from 3 to 25 Swiss francs in 1-franc steps. In half of the options, the participant’s own cost was lower than the other’s benefit, whereas in the other half, the participant’s own cost was higher than the other’s benefit. As participants always incurred a cost when they accepted an option, we defined generosity as the acceptance rate. The participants were endowed with 30 Swiss francs per hour, which they could also use to bear the costs of gifts for others. After the entire experiment was completed, one trial was randomly selected and, if it was an accept trial, the corresponding monetary benefit for the other person was sent to the chosen recipient and the participants were asked to pay the costs of the selected option. Trials were separated by a variable intertrial interval (ranging from 2 to 8 s, mean±s.e.m. 3.37±0.231 s). Importantly, the investigator who explained and conducted the decision-making task in the scanner was blind to the participant’s group membership. We contacted the participants by telephone at the end of each week and asked them how they had spent the money. Unfortunately, five participants did not respond, but otherwise all other participants (*n*=43) reported that they had spent the money according to the instructions.

### Behavioural data analysis

For each participant, generosity was defined as the mean acceptance rate, that is, the number of accept trials divided by the total number of trials with a response. We examined the effect of the manipulation by comparing the experimental group to the control group with a two-sample *t*-test. We found little evidence to suggest that distributions of acceptance rates deviated from normal distributions (Kolmogorov–Smirnov tests, *n*=24 for each group, both *P*>0.05). We tracked the increase in happiness over time by subtracting the mean pre-experimental (Time Point 1) SHS score from the mean post-experimental (Time Point 2) score. To test our specific a priori hypothesis, namely, that there would be a greater increase in happiness in the experimental group than in the control group, we assessed increase in happiness in a two-sample *t*-test (one-sided). We compared the two groups’ ratings of familiarity with and liking of the recipients using the Kolmogorov–Smirnov test. We tested the ratings for correlations with individual differences in generosity using Spearman’s rho. Each option’s net subjective value in each trial was computed as:


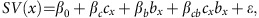


where *SV*(*x*) is the net subjective value of Option *x*, *c*_*x*_ is the cost of option *x* for self, *b*_*x*_ is the benefit of option *x* for other, and the betas (*β*) represent the participant-specific regression weights for the constant, cost, benefit and the interaction^51^. The interaction term estimates the tendency to accept (negative interaction term) or reject (positive interaction term) offers for which costs and benefits are dissimilar, such as in cases in which recipients receive a small benefit at a higher cost to participants. This model with the interaction term was selected because it has been shown that a subjective value model including the interaction term is superior in predicting brain data compared to models without the interaction term.

### Imaging data acquisition and preprocessing

Functional imaging was conducted by a Philips Achieva 3T whole-body scanner with an eight-channel sensitivity-encoding head coil (Philips Medical Systems) at the Laboratory for Social and Neural Systems Research, University of Zurich. The task was visually presented through a mirror mounted on the head coil. We acquired gradient echo T2*-weighted echo-planar images (EPIs) with BOLD contrast (slices/volume, 33; ascending order; 244 slices × 3 runs). Imaging parameters were as follows: repetition time (TR), 2 s; echo time (TE), 25 ms; flip angle, 80°; matrix size, 96 × 96; field of view, 192 mm and voxel size, 2 × 2 × 2.6 mm^3^. Functional data were preprocessed with SPM8 (Wellcome Department of Imaging Neuroscience, University College London, Institute of Neurology, London, UK). Images were slice-time corrected, realigned, spatially normalized to a standard MNI template (resampling to 3 mm isotropic voxels) and spatially smoothed with a Gaussian kernel of 8 mm FWHM.

### General activation effects of generosity commitment

To capture the general effect of the commitment to be generous during the decision task, we first set-up a general linear model (GLM) with two onset regressors, one for accept trials and one for reject trials at the time of decision. Also, six movement parameters were included as regressors of no interest. The regressors were convolved with the hemodynamic response function (HRF). Next, we computed for each participant the contrast of accept versus reject trials and assessed the group difference by taking the resulting contrast images to a second-level mixed-effect analysis using a voxel-wise two-sample *t*-test. A whole-brain correction for multiple comparisons was performed at the cluster level. The cluster-defining voxel-level threshold was *P*<0.001, together with an extent threshold of *k*≥10 voxels. All reported coordinates (*x*,*y*,*z*) are in MNI space. Furthermore, we tested for lateralization by comparing extracted signals from the regions of interest in the left and right TPJ (3-mm and 5-mm sphere around the peak voxel (−51 to 70 34) and (51 to 70 34), respectively). Here, we applied a paired *t*-test.

### Generosity-dependent TPJ connectivity modulation

We performed a whole-brain PPI analysis^21^,^22^,^52^ with the TPJ (identified in the preceding analysis) as a seed region. We extracted the entire time series over the experiment from each participant in the TPJ cluster that showed a significant group difference in accept versus reject trials. To create the PPI regressors, we multiplied the normalized TPJ BOLD time series with two condition vectors containing ones for six repetition times (TRs) from the time of decision for each decision type (one regressor for accept trials and one for reject trials) and zeroes otherwise. The method used here relies on correlations in the observed BOLD time series, thus making no assumptions about the nature of the neural events contributing to the BOLD signal^22^,^52^. The time window of six TRs (12 s) was selected to capture the first part of the HRF, which peaks after three TRs and is back at baseline at approximately eight TRs after stimulus onset.

The PPI regressors were used in a separate GLM, which included: (i) psychological regressor of accept trials, (ii) psychological regressor of reject trials, (iii) physiological regressor (the entire time series of the TPJ over the entire experiment), (iv) the PPI regressor for accept trials and (v) the PPI regressor for reject trials. The psychological regressors (i and ii) were convolved with a HRF. The parameter estimates associated with the PPI regressors (iv and v) represent the extent to which activity in each voxel of the brain correlates with activity in the TPJ during accept or reject trials. We then computed for each participant the contrast between accept versus reject PPI regressors and entered the resulting contrast images into a one-sample *t*-test together with three covariates: (a) group (coded as one or minus one for experimental or control group), (b) individual acceptance rate and (c) group × acceptance rate interaction. Covariate (c) allowed us to identify voxels where group membership and acceptance rate interacted significantly in TPJ connectivity during accept versus reject trials. Since we had a priori hypotheses about the target regions for these analyses, we performed the multiple comparison threshold corrections at the voxel level (family-wise error (FWE), *P*<0.05) within 10-mm spheres of interest that had been defined by previous publications. Rutledge *et al*.^11^ defined bilateral ventral striatum (left (−9, 8, −8) and right (18, 8, −5)) as a happiness-coding region and Kahnt *et al*.^53^ defined OFC (3, 54, −14) as a reward-coding region. The OFC has been shown to represent value during both anticipation and receipt of reward.

### Neural link between generosity and increased happiness

In the previous TPJ-PPI analysis, we identified a cluster in the ventral striatum that was modulated by specific TPJ connectivity as a function of generosity. In the next step, we investigated the happiness-related function of this region during decisions to be generous (that is, individual differences in the proportion of generous choices). We therefore related the BOLD response during the accept decisions to individual increases in happiness and investigated whether the relation was group-specific. For this analysis, we used GLM with two onset regressors, accept and reject trials, respectively (see also the section entitled ‘General activation effects of generosity commitment’). At the second level, individual contrast images for accept versus reject decisions were then entered into a one-sample *t*-test together with three covariates: (a) group (coded as one and minus one for the experimental and the control group), (b) increase in happiness and (c) group × increase in happiness interaction. Covariate (c) allowed us to identify voxels where BOLD responses reflecting increase in happiness during accept versus reject trials interacted with group membership.

Morever, to statistically test whether the striatal region representing an increase in happiness was the same striatal region in which activity is being modulated by TPJ connectivity, we performed a conjunction analysis, defined as an AND between truth statements^54^,^55^. For all conjunction analyses, we set the threshold of *P*<0.005, *k*=10 for both images. In other words, only voxels that showed significantly greater activation above this threshold in both analyses were included^54^,^55^.

### OFC coding option value

To identify regions coding subjective option value in relation to individual generosity levels (acceptance rate), we used a GLM with a trial-wise parametric modulator capturing net subjective value of the presented option for each participant. The option’s net subjective value in each trial was computed as described above. In each trial, we parametrically modulated the regressor for the presented option by the trial-wise subjective value at the time of decision. Both regressors were convolved with the HRF. The parametric modulator was then regressed against the BOLD signal in each voxel. Individual contrast images for the parametric value modulation were taken to a second-level random effect analysis using a one-sample *t*-test together with two covariates: (a) group (coded as one and minus one for experimental and control group) and (b) individual acceptance rate. We then identified significant voxels for (b). This identified regions in which the coding of subjective value correlated with individual generosity.

Finally, to test whether the OFC region modulated by TPJ connectivity is the same OFC region coding subjective value, we performed a conjunction analysis analogous to that described in the section above entitled ‘Neural link between generosity and increase in happiness’. Connectivity analyses were the same as described above for the striatum: the seed region used was the TPJ ROI and the psychological variable used was accept/reject.

### Data availability

The data that support the findings of this study are available from the corresponding author upon reasonable request.

## Additional information

**How to cite this article:** Park, S. Q. *et al*. A neural link between generosity and happiness. *Nat. Commun.* **8,** 15964 doi: 10.1038/ncomms15964 (2017).

**Publisher’s note:** Springer Nature remains neutral with regard to jurisdictional claims in published maps and institutional affiliations.

## Supplementary Material

Supplementary Information
