# Supplementary material for: A neural link between generosity and happiness
Source: Nat Commun. 2017 Jul 11;8:15964. doi: 10.1038/ncomms15964 (PMC5508200; doi:10.1038/ncomms15964)
Supplement: Supplementary Information [file ncomms15964-s1.pdf]

Title of file for HTML: Supplementary Information
Description: Supplementary Figures, Supplementary Tables and Supplementary References

Supplementary Information

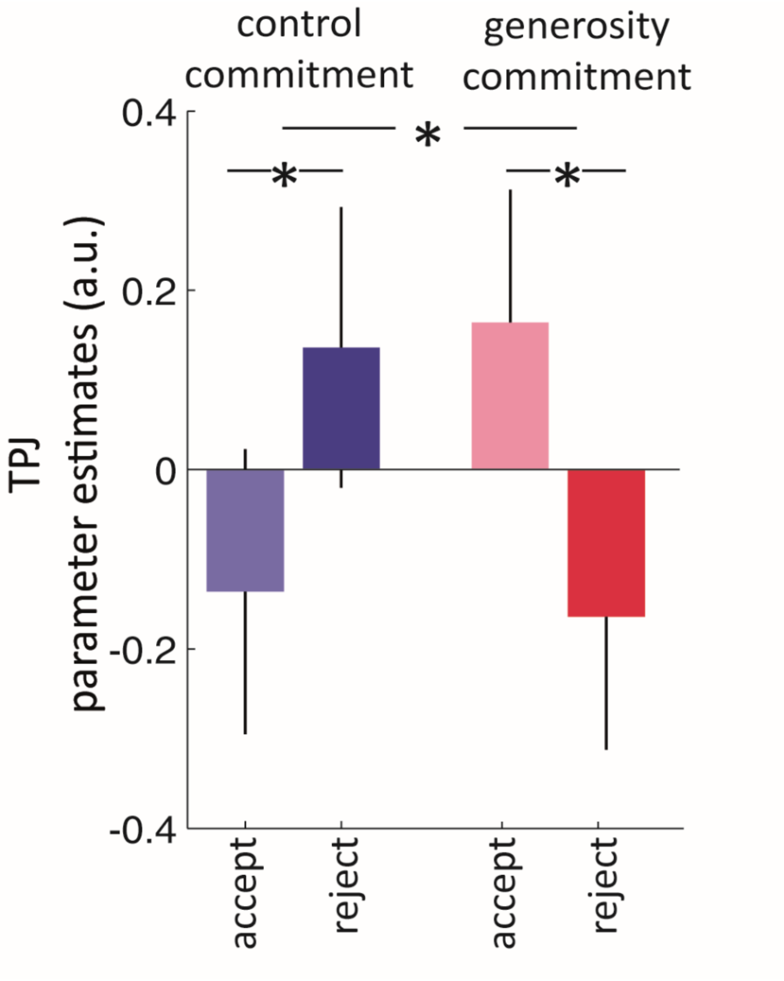

Supplementary Figure 1. The mean parameter estimates of the TPJ region showing significant group differences for accept>reject trials.

In the control group ($t(23)=3.3$; $p=0.003$) as well as in the experimental group ($t(23)=4.7$; $p<0.001$), there was a significant difference in TPJ activation in accepted vs. rejected trials. Error bars are s.e.m.

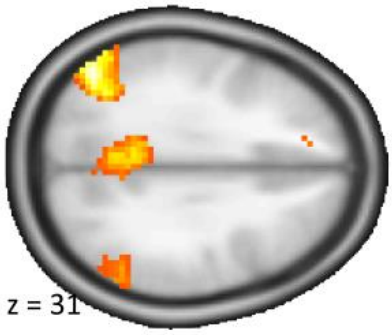

Supplementary Figure 2. There was greater activation in the left as well as the right TPJ regions in the experimental group, as compared to the control group, during accept vs. reject trials (shown at $p<0.05$, uncorrected only for demonstration purposes). However, only the left TPJ survived the threshold for multiple comparison correction.

Supplementary Table 1. The mean (and standard error of the mean) of model weights for the experimental and the control group.

	Experimental group	Control group
Coefficient own cost	0.26 (±0.09)	0.24(±0.09)
Coefficient other benefit	0.22 (±0.08)	0.22 (±0.08)
Coefficient interaction	-0.05 (±0.06)	-0.0065 (±0.08)
Constant	0.59 (0.13)	0.5 (0.18)

Supplementary Table 2. Ruling out possible alternative explanations: prosociality and empathy trait.

We tested for group differences in individuals' prosociality and empathy trait by using the interpersonal reactivity index (IRI; [1]). Due to a technical problem with the IRI, we were only able to assess a subset of items that were distributed in all 4 subscales (Items: 1-9). Although the results need to be considered with caution, we did not find any significant group differences in either overall score or in any of the subscale scores. In addition, the IRI scores did not correlate with individual differences in generosity.

	T(46)	P
SVO	-0.49	0.63
IRI - Overall score	0.82	0.42
IRI – Fantasy	1.1	0.31
IRI - Empathic concern	0.44	0.42
IRI - Perspective taking	0.47	0.64
IRI - Personal distress	0.9	0.37

Supplementary Table 3. Correlations between the individual differences in empathy and generous behavior.

r (p)	across the group	in the intervention group	in the control group
SVO	0.12 (0.44)	0.16 (0.43)	0.13 (0.54)
IRI - Overall score	0.2 (0.2)	0.08 (0.7)	0.02 (0.92)
IRI – Fantasy	0.09 (0.55)	0.25 (0.24)	0.04 (0.86)
IRI - Empathic concern	0.17 (0.23)	0.2 (0.36)	0.11 (0.6)
IRI - Perspective taking	-0.07 (0.63)	-0.12 (0.57)	0.02 (0.93)
IRI - Personal distress	-0.03 (0.85)	-0.07 (0.73)	-0.06 (0.77)

Supplementary Table 4. GLM1: Regions showing group differences (experimental group vs. control group) for generous decisions (accept vs. reject an option).

Region	Side	Cluster Size	MNI coordinates	T value
Temporo-parietal junction	L	65	-51, -70, 34	4.70
Superior frontal gyrus	L	40	-18, 41, 43	4.73
Precuneus	L	20	-6, -46, 37	3.94

Threshold: $k \geq 10$ voxels, $t(46) > 3.28$, $p < 0.001$

Supplementary Table 5. GLM2: Regions showing group differences (experimental group vs. control group) with regard to TPJ connectivity predicting individual differences in generous behavior (acceptance rate). The psychological variable was generous decisions, i.e. accept vs. reject an option.

Region	Side	Cluster Size	MNI coordinates	T value
Inferior orbitofrontal cortex	L	1163	-21, 26, -14	6.00
Middle / superior frontal orbitofrontal cortex	R	154	18, 38, -17	5.60
Striatum	R	55	21, 5, -5	4.81
Striatum	L	21	-15, 11, -5	5.07
Temporal gyrus	L	156	-27, -52, 25	5.54
Hippocampus	R	154	24, -34, 1	5.44
Middle temporal gyrus	R	30	66, -37, 1	5.01
Dorsolateral prefrontal cortex	R	27	45, 41, 31	4.92
Superior temporal gyrus	R	23	57, 11, -17	4.88
Inferior parietal lobe	L	40	-45, -61, 52	4.87
Middle frontal gyrus	L	25	-45, 35, 34	4.36
Thalamus	L	12	-12, -13, 7	4.10
Cerebellum	R	21	21, -34, -20	3.56

Threshold: $k \geq 10$ voxels, $t(44) > 3.29$, $p < 0.001$

Supplementary Table 6. GLM3: Regions showing an interaction of group (experimental group vs. control group) and change in happiness while participants made generous decisions (accept vs. reject an option).

Region	Side	Cluster size	MNI coordinates	T value
Striatum	L	29	-21, 2, -5	4.34

Threshold: $k \geq 10$ voxels, $t(44) > 3.29$, $p < 0.001$

Supplementary Table 7. Regions showing a correlation between generosity (acceptance rate) and activity in the contrast [accept vs. reject] in all participants (i.e. across groups)

Region	Side	Cluster size	MNI coordinates	T value
Middle temporal gyrus	R	17	60, -1, -17	7.75
Operculum	R	23	42, -7, 19	6.92
Cingulate gyrus	R	14	3, 8, 19	6.67
Fusiform gyrus	R	17	21, -37, -17	5.26

Threshold: $k \geq 0$ voxels, $t > 5.32$, $p < 0.05$ (FWE-corrected)

Supplementary Table 8. GLM4: Regions showing a correlation with subjective value in all participants.

Region	Side	Cluster size	MNI coordinates	T value
Medial orbitofrontal cortex	R	91	0, 62, -11	4.63
Amygdala	R	68	21, 2, -20	4.66
Amygdala	L	32	-24, 5, -20	4.42
Middle temporal gyrus	R	49	57, -4, -11	5.17
Inferior parietal lobe	R	221	63, -34, 37	5.07
Precuneus	L	61	-9, -52, 58	4.45
Precentral gyrus	R	10	27, -22, 61	4.13
Inferior parietal lobe	L	66	-66, -31, 28	4.10
Superior temporal gyrus	R	16	63, -64, 16	3.76
Middle frontal gyrus	L	15	-30, 32, 43	3.63
Cerebellum	R	44	18, -37, -14	4.73
Cerebellum	L	70	-24, -37, -14	4.66

Threshold: $k \geq 10$ voxels, $t(45) > 3.28$, $p < 0.001$

Supplementary Table 9. Regions showing a correlation between generosity (acceptance rate) and TPJ connectivity for the contrast [accept vs. reject] in all participants (i.e. across groups) .

Region	Side	Cluster size	MNI coordinates	T value
Middle frontal gyrus	L	249	-30, 53, 25	6.11
Superior temporal pole	L	25	-30, 5, -26	4.90
Anterior cingulate cortex	L	20	-12, -13, 40	4.56
Precentral gyrus	L	13	-36, -4, 55	4.44
Precentral gyrus	R	31	24, -13, 43	4.43
Superior temporal pole	R	13	33, 11, -26	4.38
Cerebellum	R	10	6, -31, -23	3.79
Superior frontal gyrus	R	12	30, 65, 4	3.60

Threshold: $k \geq 10$ voxels, $t > 3.29$, $p < 0.001$ (uncorrected)

Supplementary references

1. Davis, M. Measuring Individual Differences in Empathy: Evidence for a Mutlidimensional Approach. *J. Pers. Soc. Psychol.* **44,** 113–126 (1983).

2. The giving pledge, http://givingpledge.org/ (2017)

3. Giving what we can, https://www.givingwhatwecan.org/ (2017)
